# Effect of Ampelopsis Radix on wound healing in scalded rats

**DOI:** 10.1186/s12906-015-0751-z

**Published:** 2015-07-08

**Authors:** Kyungjin Lee, Byonghee Lee, Mi-Hwa Lee, Bumjung Kim, Khanita Suman Chinannai, Inhye Ham, Ho-Young Choi

**Affiliations:** Department of Herbology, College of Korean Medicine, Kyung Hee University, 26 Kyungheedae-ro, Dongdaemun-gu, Seoul, 130-701 Republic of Korea

**Keywords:** *Ampelopsis japonica* makino, Scald wound, Wound healing, TNF-α, VEGF

## Abstract

**Background:**

Ampelopsis Radix has been used as a traditional Korean medicine for the treatment of burns and scalds. However, there has been no scientific research to date on the wound healing properties of Ampelopsis Radix for scald burns. This study aimed to evaluate the healing effect of *Ampelopsis japonica* root tuber ethanol extract (AJE) on induced cutaneous scald injury in Sprague Dawley (SD) rats.

**Methods:**

Hot water scalds were induced in SD rats, who were then divided into the following 5 groups; 1) control group without treatment, 2) positive control group with 1 % Silver sulfadiazine (SSD), 3) Vaseline group, and groups 4) and 5) that used Vaseline containing 5 % and 20 % AJE, respectively. The ointment was applied topically to the experimental rats, once daily for 21 days, starting at 24 h post induction of the scald injury. Gross examination, measurement of wound size, and histopathological examination were performed. And quantitative measurement of cytokine levels of tumor necrosis factor alpha (TNF-α), interleukin-10 (IL-10), transforming growth factor beta 1 (TGF-β1), and vascular endothelial growth factor (VEGF) were performed by enzyme-linked immunosorbent assay.

**Results:**

Clinical evaluation showed that the AJE and Vaseline groups, rapidly desquamated scab on day 12 post-scalding; in particular, the 20 % AJE group achieved the greatest extent of skin recovery. Sizes of scald wound were significantly lower on days 12, 15, 18, and 21 in the AJE treated groups compared to the control groups. Histopathological evaluation showed a well-organized epithelial layer, angiogenesis, tissue granulation and collagen formation with the exception of inflammatory cells in the AJE-treated groups compared to the control groups on day 14, indicating that tissue regeneration had occurred. AJE treatment decreased TNF-α and increased IL-10 levels on days 2 and 14, indicating the anti-inflammatory action of AJE. The AJE groups also showed a decrease in TGF-β1 levels on day 7 and VEGF on day 14 in the serum of scald inflicted SD rat model.

**Conclusions:**

These results suggest that AJE possesses scald wound healing activity via accelerating the scald wound repair during the inflammation and proliferative phases of the healing process.

## Background

Recently, health care professionals have been faced with an increasing number of patients suffering from wounds and burns [1]. Chronic, non-healing wounds and their treatment represent a major medical and economic problem [2, 3]. The demand for natural remedies is growing in developing countries because they are safe, cheap and useful to treat burn injury [4–7]. The use of traditional remedies and plants in treating burns and wounds is an important mode to improve healing, as well as to reduce the financial burdens of treatment. Several plants have been used as a traditional medicine to treat skin disorders and injuries [4, 7–10].

Ampelopsis Radix is the dried root tuber of *Ampelopsis japonica* Makino (Family Vitaceae). The actions of Ampelopsis Radix are to clear heat and eliminate toxins; disperse abscesses and dissipate binds; and to promote wound healing and tissue regeneration. Indications for Ampelopsis Radix are abscesses, cellulitis, carbuncles of the back, deep-rooted boils and sores, scrofula, burns, and scalds [11–13]. Recently, external application of Ampelopsis Radix has been reported to have wound healing effect on 80 cases of second degree burn injury in china [14], on scald model of the mice and rats [15], and on sores and ulcers rat model [16]. As mentioned above, Ampelopsis Radix has been used to treat burns and scalds in traditional medicine and reported to have wound healing effects, but no study has yet reported the mechanisms of action of Ampelopsis Radix on burns and scalds.

In the present study, the healing effects and mechanisms of action of the ethanol extract of *A. japonica* Makino root tuber (AJE) on scald wounds using an experimental rat model were investigated.

## Methods

### Plant material and extraction

Dried tuberous root of *Ampelopsis japonica* Makino (AJ) was purchased from an herbal drug company, DongWooDang Pharmacy Co., Ltd. (Yeongchen, Gyeongsangbuk-do Province, Korea). It was identified by Professor Youngmin Bu. AJ (voucher specimen No. AJ 001) used in this study was deposited in the Laboratory of Herbology, College of Korean Medicine, Kyung Hee University, Seoul, Korea.

AJ (500 g) was extracted three times for 3 h with 100 % ethanol under heating mantle-reflux. The extract was then condensed with a rotary vacuum evaporator (N-N series, Eyela Co., Japan). The yield of crude extract was 5.12 %.

### Reagents and equipment

Veet hair removal cream was purchased from Reckitt Benckiser (France). White Vaseline was purchased from Korea-ione Co. Ltd. (Gyeonggi-Do, Korea). Silmazin 1 % cream was purchased from Dong-Wha Pharm. Co. Ltd. (Seoul, Korea). Rat interleukin-10 (IL-10) enzyme-linked immunosorbent assay (ELISA) kits and rat transforming growth factor beta 1 (TGF-β1) ELISA kits were purchased from Cusabio Biotech Co. Ltd. (Wuhan, Hubei Province, China). Rat tumor necrosis factor alpha (TNF-α) ELISA kits and rat vascular endothelial growth factor (VEGF) ELISA kits were purchased from Koma Biotech Inc. (Seoul, Korea). The Masson-Goldner trichrome staining kit was purchased from Merck in south Korea (Seoul, Korea), and hematoxylin, eosin Y alcoholic, and Acid Alcohol · Histo^TM^ were purchased from BBC Biochemical Co. (USA). Ammonium hydroxide ACS reagent was purchased from Sigma-Aldrich Co. Inc. (USA), and Harris ethyl alcohol and xylene were purchased from J.T.Baker^Ⓡ^ (Japan).

In the present study a rotary evaporator (Eyela Co., Japan), ELISA Plate Reader: VersaMax (Molecular Devices Co., USA), digital camera (Sony Corporation, Japan), micro high speed centrifuge (Vision Scientific Co. Ltd., Korea), HM440E microtome (Carl Zeiss, Germany), Olympus DP70 digital microscope camera and Olympus DP controller software (Olympus Imaging America Inc., USA) were used.

### Animals

To examine the scald-healing effects of AJE, tests were performed on 6-week-old male Sprague–Dawley (SD) rats (weight, 180–220 g; Samtaco, Korea). The rats were housed under controlled conditions (22 ± 2 °C; lighting, 07:00–19:00) at a pathogen-free animal facility at Kyung Hee University. Food and water were available *ad libitum*. The experiments were conducted according to the guidelines presented by the Committee for Animal Care and Use of Laboratory Animals, College of Korean Medicine, Kyung Hee University (protocol approval number KHUASP(SE)-13-003).

### Scald wound induction and treatment

To examine the effects of the AJE on the scald wound healing process, scald wounds were induced on the backs of SD rats under anesthesia with ethyl ether. Their dorsal hair was shaved and the residual hair was removed with depilatory cream. After shaving, the dorsal surface was wiped with warm distilled water and 70 % ethanol. The scald wounds were created using the method described by Stevenson et al. [17] with some modification. Hot water induced the second degree scald wound (100 °C for 10 s) on the dorsal surface in approximate diameter 2.8 cm. After scalding, rats were assigned at random to five groups (Control, Silver sulfadiazine, Vaseline, 5 % and 20 % AJE, *n* = 8, respectively) and after 24 h, 0.5 g of the test substance was applied topically to the scald area once daily for 3 weeks as follows: Control (CON), non-treated rats after scald; Silver Sulfadiazine (SSD), wounds treated with reference standard a 1 % (w/w) SSD cream (*n* = 8); Vaseline, wounds treated Vaseline; 5 % and 20 % AJE, wounds treated with a Vaseline-based 5 and 20 % (w/w) AJE ointment.

### Gross examination of the scald wound lesions

The wounds were grossly examined on day zero following the scald injury, and then at three-day intervals. The lesions were examined using the following criteria: wound bed color, exudates, swelling of the wound surface, and consistency of tissues surrounding the wound. Immediately after inducing the scald wound, the wound area was measured and a wound picture/image was captured using a digital camera. Wound pictures were taken every 3 days and analyzed using ImageJ (Broken Symmetry Software). The percentage wound contracture rate was calculated using following formula: % Contracture = Specific day wound size / Initial wound size × 100

### Collection and processing of serum and scald wound tissue

Blood samples from each group were collected from the tail vein in non-coated tubes, at 2, 7, 14, and 21 days after the scald injury. Collected samples were centrifuged at 8,000 rpm for 40 min, and serum was separated and stored at −80 °C until analysis. Scald wounds were excised down to the level of the muscle fascia by sharp dissection and included the surrounding wound margin tissue [18].

### Analysis of cytokine and angiogenesis factors

Serum samples were quantitatively assayed for the inflammatory cytokines TNF-α and IL-10 and the angiogenesis factors TGF-β1 and VEGF using ELISA systems. Measurements were performed according to the manufacturer’s instructions for TNF-α, IL-10, TGF-β1, and VEGF using a 96-well microplate reader at 450 nm. All samples for each cytokine were used in triplicate, and optical density measurements were then verified against a standardized curve. The results were averaged and expressed as pg/mL or ng/mL.

### Histopathological studies with hematoxylin and eosin and Masson-Goldner trichrome stain

Rats were sacrificed at days 2, 14, and 21 post-scald using ether, and skin samples were taken for histopathological examination. The skin samples were fixed in 10 % formalin solution. After fixation, the tissues were washed in running tap water, dehydrated in ascending grades of ethyl alcohol, and cleared in xylene. Paraffin embedded tissue sections of 6-μm thickness were cut using a microtome and mounted on glass slides. Histological sections were stained with hematoxylin and Eosin (H&E) and Masson-Goldner trichrome for histological study. Digital photomicrographs were captured at representative locations using a digital camera attached to a microscope. Masson’s trichrome stains collagen blue, while cytoplasm, red blood cells, and muscle are stained red; it is typically used to assess the advancement of collagen deposition during the formation of granulation tissue and matrix remodeling [19]. The blue color staining intensity corresponds to the relative quantity of collagen fiber deposited, which reflects the process of synthesis, degradation, and remodeling of tissue [20]. Tissue samples were evaluated for the following histological criteria: extent of re-epithelialization, maturation and organization of the epidermis, granulation tissue formation, collagenization, and inflammatory cells and scar formation in the dermis. The sections were evaluated using a scoring scale of 0–3 in five categories (epithelialization, vascularization, inflammatory cell response, occurrence of granulation tissue, and collagen deposition), which provided the total graded on a scale of 1–4 [21, 22].

### Preliminary phytochemical screening

Ethanol extract of AJ is usually used in traditional Korean Medicine to treat several skin diseases and several qualitative and quantitative studies were performed using ethanol or methanol extract of AJ [23–27].

### Statistical analysis

Data are expressed as mean ± standard error of the mean (SEM). All the statistical comparisons were made using one-way analysis of variance (ANOVA) followed by the Tukey’s post-hoc test with SPSS v.13.0 statistical analysis software (SPSS Inc., USA). P values less than 0.05 were considered statistically significant.

## Results and discussion

### Gross examination

**Fig. 1:**
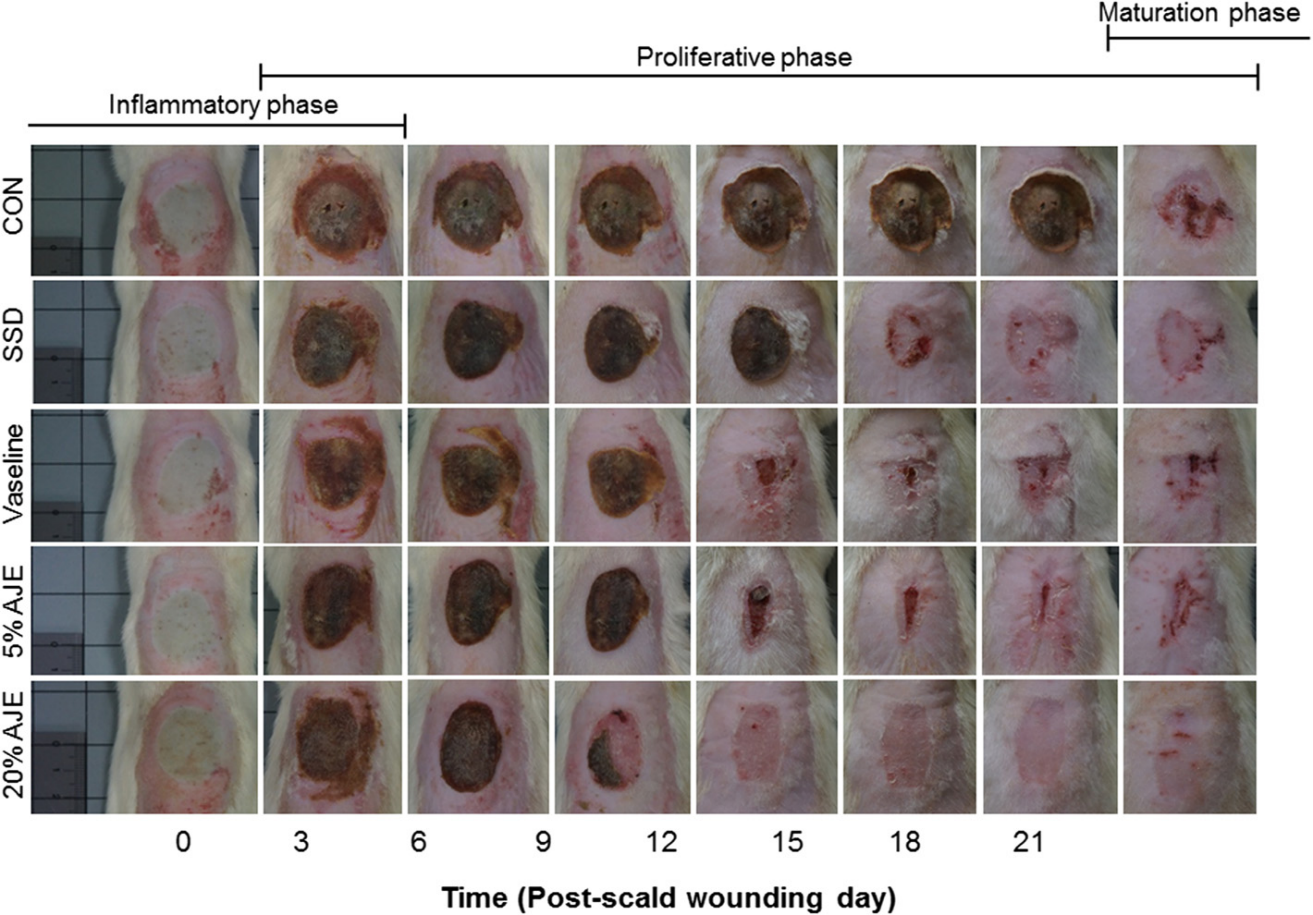
Gross appearance of the scald wounds. Abbreviations: CON, control; SSD, silver sulfadiazine; AJE, *Ampelopsis japonica* tuberous root ethanol extract

Observations of any changes in the overall appearance of the wounds took place on days 0, 3, 6, 9, 12, 15, 18, and 21. From day 3 to day 9, the ointment-treated groups exhibited yellowish-brown, moist, soft, and supple scabs with red rims along the margins. After day 12, the Vaseline and AJE treated group did not exhibit thick scabs and bleeding, whereas the control and SSD treated group showed thick, dry, and dark brown scabs that were intact. By day 15, re-epithelialization was observed in all ointment-treated groups but the control group. Wounds healed best in the 20 % CGE treated group after day 12: the wounds nearly healed, while the wound of control and SSD-treated group still exhibited a dry appearance with dark brown scab (Fig. 1).

### Measurement of scald wound size

**Fig. 2:**
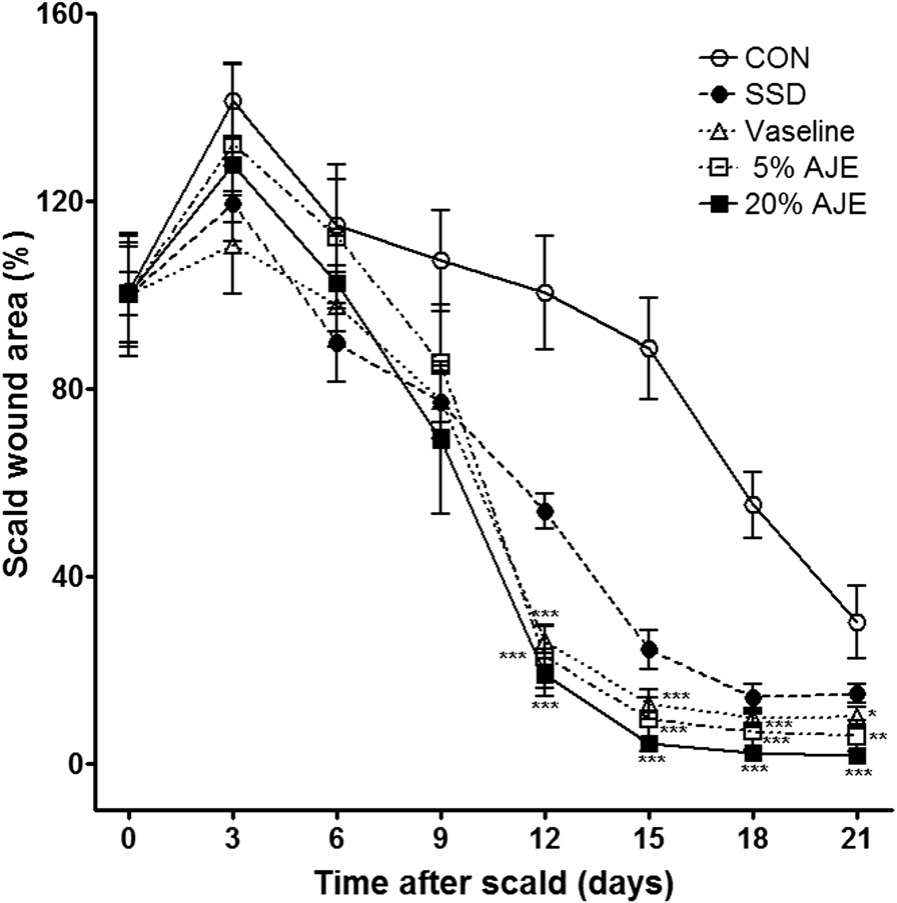
Changes in scald wound sizes. The percentage wound contracture rate was calculated using following formula: % Contracture = Specific day wound size / Initial wound size × 100 Abbreviations: CON, control; SSD, silver sulfadiazine; AJE, *Ampelopsis japonica* tuberous root ethanol extract. Values expressed as mean ± standard error of the mean (*n* = 5–8). ^*^ *P* < 0.05, ^**^ *P* < 0.01, and ^***^ *P* < 0.001 *vs*. control

In the first 0 to 9 days, there was nearly no difference between ointment-treated groups and control group in the wound contraction ratio. On day 12, the healing rates in all ointment-treated groups were significantly higher than that of the control group. On day 12, the 20 % AJE group showed the best healing rate with a wound size of 19.0 ± 4.6 %. AJE groups showed the lowest mean size of wound area from day 12 to day 21 (Fig. 2).

### Histopathological examination

**Fig. 3:**
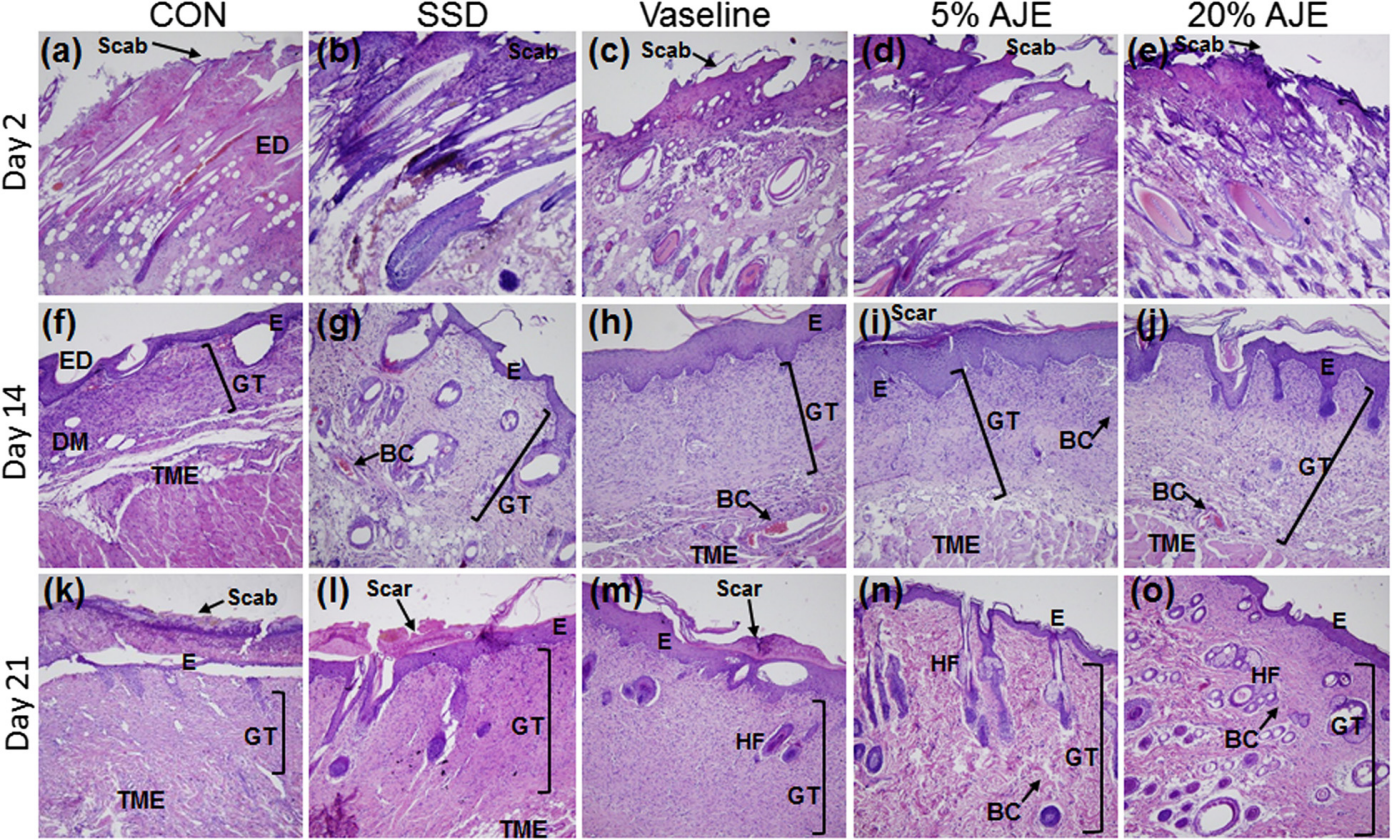
Histological appearance of scald wounds stained with hematoxylin and eosin [(**a**), (**b**), (**c**), (**d**), (**e**), (**f**), (**g**), (**h**), (**i**), (**j**), (**k**), (**l**), (**m**), (**n**) and (**o**)]. The AJE-treated groups seemed to be better engaged in the wound repair processes (re-epithelialization, vascularization, inflammation, and development of granulation tissue). All treated groups showed sufficient vascularization. Significant masses of granulation tissue could be observed in the AJE-treated group on day 21 [(**n**), (**o**)]. Among them, the thickest granulation tissue was observed in 20 % AJE group on day 14 (**j**). Magnification: ×100. Abbreviations: CON, control; SSD, silver sulfadiazine; AJE, *Ampelopsis japonica* root ethanol extract; DM, dermis; ED, epidermis; HF, hair follicle; E, epithelialization; GT, granulation tissue; BC, blood capillaries; TME, tunica muscularis externa

**Fig. 4:**
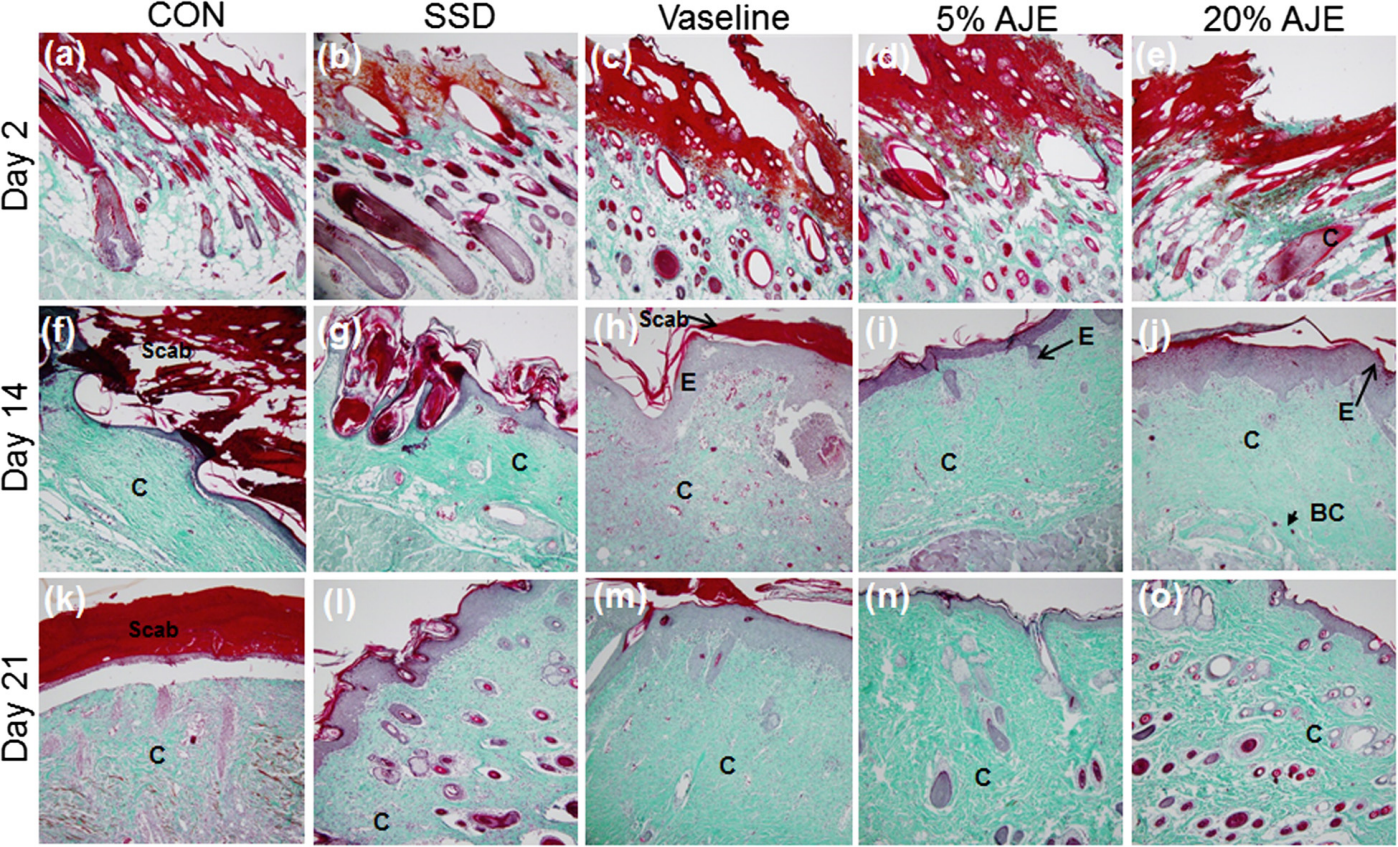
Histological appearance of scald wounds stained with Masson-Goldner trichrome stain  [(**a**), (**b**), (**c**), (**d**), (**e**), (**f**), (**g**), (**h**), (**i**), (**j**), (**k**), (**l**), (**m**), (**n**) and (**o**)]. Collagen deposition occurred more efficiently in the AJE-treated groups compared to the control group. The thick regularly aligned collagen fiber was observed and the thick scab had disappeared in AJE-treated groups on the 14 day post-scald [(**i**), (**j**)]. Vaseline group and 5 % AJE group was observed well organized collagen on the 21 day [(**m**), (**n**)]. Magnification: ×100. Abbreviations: CON, control; SSD, silver sulfadiazine; AJE, *Ampelopsis japonica* root ethanol extract; C, collagen; E, epithelialization; BC, blood capillaries

Histopathological severity evaluation revealed that all of the scald wounds were in the deep second degree category. 1) Epithelialization: Epidermal regeneration was not observed on day 2. On day 14, the occurrence of epithelialization (epithelialization score) was better than the day 2. The score was significantly greater in AJE treated group lesions than in control group. Epithelialization was observed rapidly ongoing in all groups except for the control group. On day 14, 21, epithelialization scores were significantly higher in 5 % and 20 % AJE treated group than in the control group. 2) Vascularization: Vascularization was already occurred in Vaseline and AJE treated groups on day 2. On the other hand, vascularization did not yet occurred in control and SSD treated group on day 2. On day 14, vascularization scores were increased in all groups. There were increased dense blood vessels distributed deeply in the tissue. On day 21, vascularization score was highest in AJE treated groups and increased slightly more on day 14. 3) Granulation: On day 2, in all scald wound injured groups, there were damage of epidermis, dermis and subcutaneous tissue were observed in the scalded region. The granulation tissue obtained from topically treated rats showed an increase in number of fibroblasts. On day 14, a little-advanced organization of granulation tissue has begun forming in the dermis. Among them, 20 % AJE group was formed most thick of the granulation tissue area. In contrast, the control group had observed irregularly forming granulation tissue. On day 21, the most significant increase in the granulation tissue was observed in ointment treated groups than control group. 4) Inflammation: On day 2, inflammatory cell infiltration was significantly increased in the all groups. On day 14, the control group exhibited wide area of ulcerations containing inflammatory cell, mild degrees of inflammation. 5 % AJE and 20 % AJE treated group showed mild inflammation. On day 21, the inflammatory cells were decreased in ointment treated groups. 5) Collagen deposition: On day 2, the collagen deposition was observed in all groups. On day 14, new formed collagen appeared abundant in the dermis. On day 21, the collagen was observed in the dermis in AJE groups, while collagen fibers filled the dermis in the control group. Collagen regeneration was significantly increased in the AJE groups at on day 14 and 21. 6) Total histopathology score: Total histopathological evaluation scores in ointment treated groups were significantly better than control group. Further, total histopathological evaluation scores in the AJE groups were significantly better than Vaseline group (Table 1, Figs. 3 and 4).

**Table 1:** Comparison of histopathological scores among the groups

Day	Epithelialization				
	CON	SSD	Vaseline	5 % AJE	20 % AJE
2	0.0 ± 0.0	0.0 ± 0.0	0.3 ± 0.3	0. 00 ± 0.00	0.1 ± 0.1
14	1.5 ± 0.4	2.3 ± 0.07	2.9 ± 0.1^**^	2.9 ± 0.1^***^	3.0 ± 0.0^**^
21	1.0 ± 0.0	2.7 ± 0.18^***^	2.9 ± 0.1^***^	2.9 ± 0.1^***^	3.0 ± 0.0^***^
	Vascularization				
2	0.0 ± 0.0	0.0 ± 0.0^#^	1.6 ± 0.6^*^	1.8 ± 0.3^*^	1.6 ± 0.4^*^
14	1.2 ± 0.4	1.7 ± 0.4	1.9 ± 0.1	2.2 ± 0.2	2.2 ± 0.2
21	1.5 ± 0.2	1.7 ± 0.1	1.9 ± 0.2	2.3 ± 0.3	2.5 ± 0.1^*^
	Granulation				
2	0.0 ± 0.0	0.6 ± 0.1^*^	1.2 ± 0.0^***^	1.5 ± 0.0^***^	1.3 ± 0.1^***^
14	1.4 ± 0.2	1.9 ± 0.1	2.0 ± 0.1^*^	2.6 ± 0.1^***,#^	2.8 ± 0.2^***,#^
21	2.8 ± 0.0	2.8 ± 0.1	2.9 ± 0.1	2.9 ± 0.1	3.0 ± 0.1
	Inflammation				
2	2.6 ± 0.2	2.0 ± 0.1	2.9 ± 0.1	2.8 ± 0.2	2.7 ± 0.1
14	2.9 ± 0.1	2.3 ± 0.2	2.3 ± 0.1^*^	1.3 ± 0.2^***,###^	1.2 ± 0.1^***, ##^
21	2.3 ± 0.3	1.3 ± 0.1^*^	1.3 ± 0.1^*^	0.9 ± 0.1^**^	0.9 ± 0.3^**^
	Collagen deposition				
2	1.2 ± 0.1	1.1 ± 0.2	1.5 ± 0.1	1.3 ± 0.2	1.1 ± 0.2
14	2.2 ± 0.2	2.5 ± 0.2	2.7 ± 0.1	2.8 ± 0.1^*^	2.9 ± 0.1^**^
21	2.3 ± 0.2	2.6 ± 0.1	2.7 ± 0.2	2.9 ± 0.1^*^	3.0 ± 0.1^*^
	Total score				
2	1.0 ± 0.0	1.4 ± 0.1	1.2 ± 0.0	1.4 ± 0.1	1.5 ± 0.2
14	2.1 ± 0.1	2.5 ± 0.1	2.7 ± 0.1^**^	3.3 ± 0.2^***, #^	3.0 ± 0.1^***^
21	2.4 ± 0.0	3.2 ± 0.1^**^	3.3 ± 0.1^***^	3.6 ± 0.1^***^	3.9 ± 0.1^***, # #^

Abbreviations: *CON* control, *SSD* silver sulfadiazine, *AJE Ampelopsis japonica* tuberous root ethanol extract. Values are expressed as mean ± standard error of the mean (*n* = 3–8). ^*^ *P* < 0.05, ^**^ *P* < 0.01, ^***^ *P* < 0.001 *vs*. Control, ^#^ *P* < 0.05, ^##^ *P* < 0.01, ^###^ *P *< 0.001 * vs*. Vaseline

### Quantification of TNF-α and IL-10

**Fig. 5:**
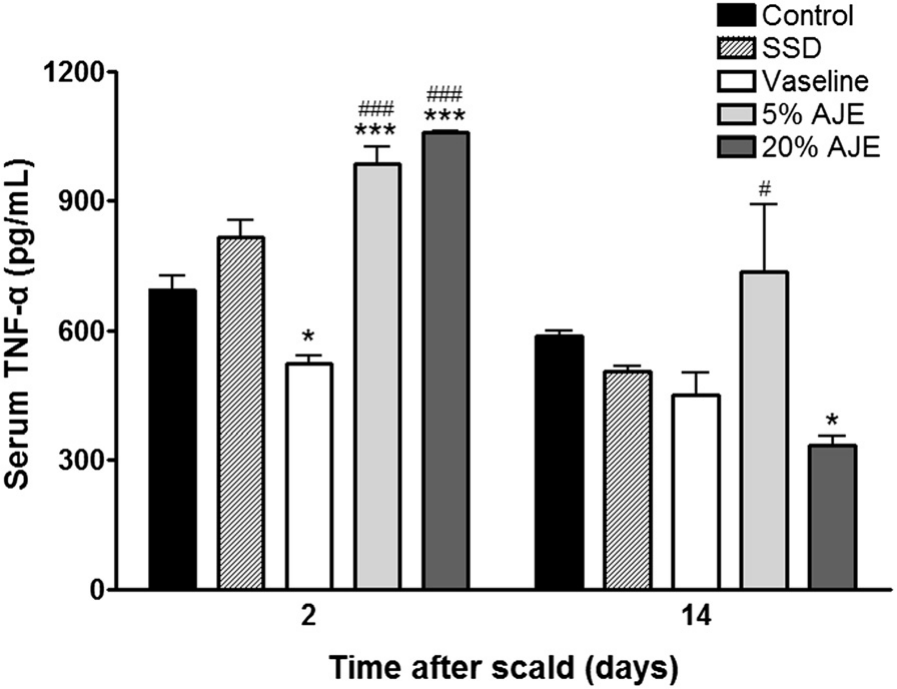
TNF-α levels in cutaneous scald injury. Abbreviations: CON, control; SSD, silver sulfadiazine; AJE, *Ampelopsis japonica* tuberous root ethanol extract; TNF-α, tumor necrosis factor alpha. Values expressed as mean ± standard error of the mean (*n* = 4–8). ^*^ *P* < 0.05, ^***^ *P* < 0.001 *vs*. control. ^#^ *P* < 0.05, ^###^ *P* < 0.001 *vs*. Vaseline

**Fig. 6:**
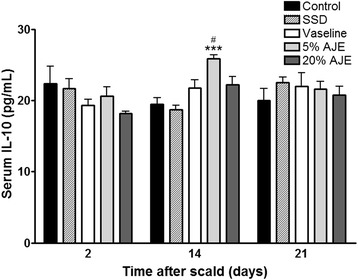
IL-10 levels in cutaneous scald injury. Abbreviations: CON, control; SSD, silver sulfadiazine; AJE, *Ampelopsis japonica* tuberous root ethanol extract; IL-10, interleukin-10. Values expressed as mean ± standard error of the mean (*n* = 4–8). ^***^ *P* < 0.001 *vs*. control. ^#^ *P* < 0.05 *vs*. Vaseline

TNF-α, a pro-inflammatory cytokine, is up-regulated during the inflammatory phase of wound healing [28] and appears to be involved in initiating the early wound healing response [29]. Low levels of TNF-α can promote wound healing indirectly but high levels of TNF-α can delay wound healing [28]. Therefore up-regulated TNF-α during the inflammatory phase should decreased for the rapid wound healing. IL-10 is an anti-inflammatory cytokine produced by various cells, including macrophages and T-lymphocytes, and is also involved in angiogenesis [30]. It is known to be a major regulator in suppressing the inflammatory response. IL-10 inhibits the synthesis of pro-inflammatory cytokines such as IL-1B, IL-6 and TNF-α in activated macrophages [31]. In the present study, serum samples were collected on days 2 and 14 to determine the impact of the scald wound on the pro-inflammatory response. On day 2, the levels of TNF-α were higher in the 5 % and 20 % AJE treated group compared to the control group and Vaseline treated group. On day 14, TNF-α level was decreased in all groups and TNF-α level was lower in the 20 % AJE treated group than Vaseline treated group (Fig. 5). On the other hand, the IL-10 levels were higher on day 14 in AJE treated groups than in the control group and Vaseline treated group. On day 21, all experimental groups were maintained higher levels of IL-10 than the control group throughout the proliferative phase (Fig. 6). In addition, the histopathological sections of the dorsal skin on day 14 in the post-scald AJE treatment groups showed a lower number of inflammatory cells than the control group (Table 1). These result suggested that AJE can reduce wound size and promote wound healing via decreasing of up-regulated TNF-α levels and Increasing of IL-10.

### Quantification of TGF-β1

**Fig. 7:**
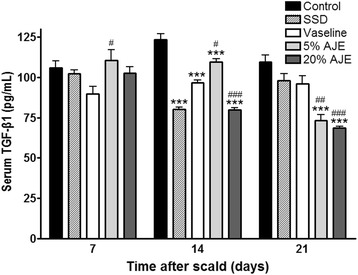
TGF-β1 levels in cutaneous scald injury. Abbreviations: CON, control; SSD, silver sulfadiazine; AJE, *Ampelopsis japonica* tuberous root ethanol extract; TGF-β1, transforming growth factor beta 1. Values expressed as mean ± standard error of the mean (*n* = 5–8). ^***^ *P* < 0.001 *vs*. control. ^#^ *P* < 0.05, ^##^ *P* < 0.01, and ^###^ *P* < 0.001 *vs*. Vaseline

TGF-β1 is a key growth factor secreted by several cells and is involved in a number of processes in wound healing, i.e., inflammation, angiogenesis, fibroblast proliferation, collagen synthesis, and remodeling of new extracellular matrix [32, 33]. Increased production of TGF-β1 supports faster re-epithelialization but hypertrophic scarring and keloid formation are occurred by over-expression of TGF-β1 during the late stages of wound healing [34]. Therefore, TGF-β1 should decreased after re-epithelialization for the wound healing without scar. In the present study, the levels of TGF-β1 were increased in all groups and decreased after 7 to 21 days in the AJE treated groups. On day 14, TGF-β1 levels in all experimental groups were lower than control group. Further, TGF-β1 level in 20 % AJE treated group was lower than Vaseline treated group. On day 21, TNF-β1 levels were significantly lower in AJE-treated groups than control group and Vaseline treated group (Fig. 7). These results supported that the scabs were removed from the AJE treated groups, but the scabs in the control group were still present on histopathological observation and general evaluation (Figs. 1, 2, and 3). Additionally, the ability of TGF-β1 to stimulate collagen production is so potent that it can result in significant changes in histopathology on AJE-treatment.

### Quantification of VEGF

**Fig. 8:**
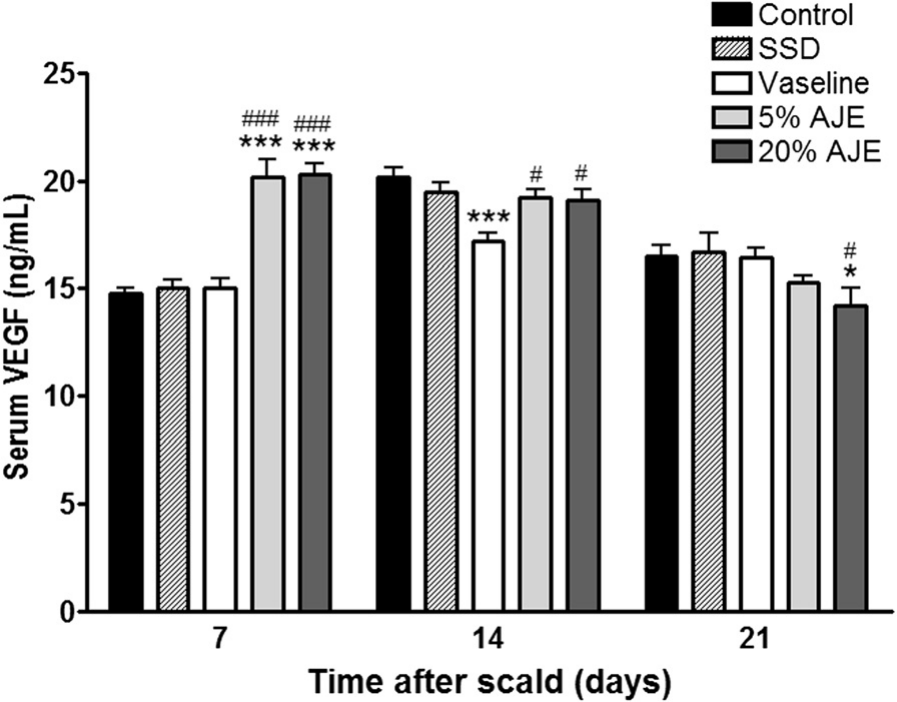
VEGF levels in cutaneous scald injury. Abbreviations: CON, control; SSD, silver sulfadiazine; AJE, *Ampelopsis japonica* tuberous root ethanol extract; VEGF, vascular endothelial growth factor. Values expressed as mean ± standard error of the mean (*n* = 5–8). ^***^ *P* < 0.001 *vs*. control. ^#^ *P* < 0.05, ^###^ *P* < 0.001 *vs*. Vaseline

Angiogenesis is an important factor in proliferative phase of wound healing. VEGF is one of the most potent proangiogenic growth factors in the skin [35]. In the last stage of wound healing, VEGF plays a role of promoting scar formation [36]. In the present study, AJE treated groups showed higher levels of VEGF than the other groups on day 7. On 14 day, the VEGF levels of control, SSD, and Vaseline treated groups were increased. On the other hand, VEGF levels of AJE treated groups were decreased. On day 21, all experimental groups were decreased in production of VEGF. Especially, VEGF levels of AJE treated groups were lower than the other groups (Fig. 8). In addition, after histopathological scoring with H&E staining, the AJE treated groups showed an increase in re-epithelialization and neovascularization compared to the Vaseline treated group and the deposition of collagen in the AJE treated group was greater than in the other groups on day 14 (Figs. 3 and 4). These results suggested that AJE could heal scald wounds faster and result in less scarring than other treatments by regulating VEGF in the whole wound healing process.

### Preliminary phytochemical screening

Preliminary phytochemical screening of AJE showed the presence of catechin, epicatechin, resveratrol, schizandriside, gallocatechin, and epicatechin gallate [24, 25, 37]. Catechin and resveratrol are the main costituents of AJE [24] and catechin was present at high concentrations in AJE [37]. Catechin [38, 39] and resveratrol [23, 40, 41] are well known anti-inflammatory and wound-healing compounds. Additionally, epicatechin, epicatechin gallate, and gallocatechin [39, 42, 43] are reported to have wound-healing properties. Therefore, the beneficial effects of AJE might be mainly attributable to catechin and resveratrol; other known and unknown compounds in AJE also might contribute to its effects.

## Conclusions

In conclusion, AJE showed faster and more effective wound healing activities than SSD and Vaseline in the skin of experimentally scalded rats. Histopathological evaluation results showed better re-epithelialization, vascularization, granulation tissue formation, and collagen deposition in the AJE treated groups than the other groups. These effects were due to the appropriate regulation of TNF-α, IL-10, TGF-β1, and VEGF. AJE could be of beneficial use in wound healing of scald injury.

## Abbreviations

AJE: *A. japonica* root tuber ethanol extract
SSD: 1 % Silver sulfadiazine
TNF-α: Tumor necrosis factor alpha
IL-10: Interleukin-10
TGF-β1: Transforming growth factor beta 1
VEGF: Vascular endothelial growth factor
AJ: Dried tuberous root of *Ampelopsis japonica* Makino
